# Insulin renders diabetic rats resistant to acute ischemic stroke by arresting nitric oxide reaction with superoxide to form peroxynitrite

**DOI:** 10.1186/s12929-014-0092-0

**Published:** 2014-09-16

**Authors:** Li-Man Hung, Jiung-Pang Huang, Jiuan-Miaw Liao, Meng-Hsuan Yang, Dai-Er Li, Yuan-Ji Day, Shiang-Suo Huang

**Affiliations:** Department and Graduate Institute of Biomedical Sciences and Healthy Aging Research Center, College of Medicine, Chang Gung University, Tao-Yuan, Taiwan; Department of Physiology, Chung Shan Medical University and Chung Shan Medical University Hospital, Taichung, Taiwan; Department of Anesthesiology, Chang Gung Memorial Hospital and Graduate Institute of Clinical Medical Sciences, Chang Gung University, Tao-Yuan, Taiwan; Department of Pharmacology and Institute of Medicine, Chung Shan Medical University and Department of Pharmacy, Chung Shan Medical University Hospital, No.110, Sec. 1, Jianguo N. Rd, Taichung City, 402 Taiwan

**Keywords:** Diabetes, Ischemic stroke, Insulin, Nitric oxide, Superoxide, Peroxynitrite

## Abstract

**Background:**

The functions of free radicals on the effects of insulin that result in protection against cerebral ischemic insult in diabetes remain undefined. This present study aims to explain the contradiction among nitric oxide (NO)/superoxide/peroxynitrite of insulin in amelioration of focal cerebral ischemia–reperfusion (FC I/R) injury in streptozotocin (STZ)-diabetic rats and to delineate the underlying mechanisms. Long-Evans male rats were divided into three groups (age-matched controls, diabetic, and diabetic treated with insulin) with or without being subjected to FC I/R injury.

**Results:**

Hyperglycemia exacerbated microvascular functions, increased cerebral NO production, and aggravated FC I/R-induced cerebral infarction and neurological deficits. Parallel with hypoglycemic effects, insulin improved microvascular functions and attenuated FC I/R injury in STZ-diabetic rats. Diabetes decreased the efficacy of NO and superoxide production, but NO and superoxide easily formed peroxynitrite in diabetic rats after FC I/R injury. Insulin treatment significantly rescued the phenomenon.

**Conclusions:**

These results suggest that insulin renders diabetic rats resistant to acute ischemic stroke by arresting NO reaction with superoxide to form peroxynitrite.

## Background

The principal mediator of cerebral injury secondary to ischemic stroke is oxidative stress [1]. Oxidative stress-mediated cerebral injury is the consequence of an imbalance between free radical generation and elimination due to increased reactive oxygen and nitrogen species generation and inadequate antioxidant defense [1]. Central nervous system cells are more vulnerable to oxidative stress given their inherent higher oxidative metabolism and less antioxidant enzymes and higher membranous fatty acid content. During ischemic stroke, the free radicals concentration rises from normal low levels to a peak point during reperfusion, possibly underlying apoptosis or cellular necrosis. Malinski et al. found that the basal nitric oxide (NO) levels are in the nanomolar range, nevertheless, the amount rapidly increases to micromolar levels following cerebral ischemia [1,2]. The cerebral ischemia reperfusion injury can also increase superoxide production [3]. In addition, increasing evidence in both experimental and clinical studies also suggests that oxidative stress is critical in the pathogenesis of both types of diabetes mellitus [4]. However, studies of diabetic NO levels have yielded conflicting results. Some investigators have demonstrated impaired NO production in diabetes [5,6]. By contrast, other studies have observed increased NO in diabetes [7] or no change in NO level in the diabetic state [8].

In the basic studies, insulin treatment protects neurological function following cerebral ischemia in hyperglycemic rats [9,10]. However, the roles of free radicals on the effects of insulin that result in protection against cerebral ischemic insult in diabetes remain undefined. The primary aim of the present study is to resolve the discrepancy of expression of NO in diabetes. The secondary aim is to explore the possible mechanisms (with specific focus on NO/superoxide/peroxynitrite) of insulin in amelioration of focal cerebral ischemia-reperfusion (FC I/R) injury in streptozotocin (STZ)-diabetic rats.

## Methods

### Experimental animals

Healthy adult male Long-Evans rats (National Lab. Animal Breeding and Research Center) were used throughout this study and were maintained in the Animal Center of the Chang Gung University, at an ambient temperature of 25 ± 1°C and a light-dark period of 12 h. The animals were fed with normal chow and water. This investigation abides by the rules in the Guide for the Care and Use of Laboratory Animals published by the US National Institutes of Health (NIH publication no. 85-23, revised 1996).

### Induction of STZ-diabetic animal model

Diabetes was induced according to the previous-described protocol [11]. The experimental animals were randomly divided into two groups: (1) age-matched non-diabetic control (Con) and (2) STZ-induced diabetic rats (DM). The animals were then injected with a single intravenous injection of vehicle (normal saline) or freshly prepared STZ (65 mg/kg). The STZ-diabetic animals were further randomly divided into two groups: (1) DM and (2) diabetic rats treated with insulin (DMI; Monotard® HM; Novo Nordisk, Bagsvaerd, Denmark).STZ-diabetic rats were subcutaneously injected with insulin (4 IU/rat/day) for 1 week (at 8 to 9 AM from days 15 to 21 of STZ injection). The animals were subjected to FC I/R injury at 8 AM on day 22.

### Surgical procedure and analysis of infarct volume

Focal ischemic infarcts were carried out by operation as reported previously with some modifications [12]. In brief, rats weighing between 250 g and 280 g were anesthetized with chlorohydrate (4.5 mg/kg, intraperitoneal injection) after 24 h of the last vehicle (normal saline) or insulin treatment. Rectal temperature was monitored and kept constant (37 ± 0.5°C) during the surgical procedure and recovery period. The animals were performed craniotomy at the junction of the zygoma and squamosal bone using a drill (Dremel Multipro + 5395, Dremel com. USA) and the dura was opened with fine forceps using a dissecting microscope (OPMI-1, ZISS, Germany). The right middle cerebral artery (MCA) was ligated with 10-0 monofilament nylon ties and both common carotid arteries were then occluded by microaneurysm clips for 1 h. After removing the clips, return of blood flow was visualized in both common carotid arteries. However, the right MCA ligature was left in place permanently [13].

Animals were sacrificed by rapid decapitation under deep anesthesia. Brains were rapidly removed and sliced into 2-mm thick coronal sections using a brain matrix slicer (JACOBOWITZ Systems, Zivic-Miller Laboratories INC, Allison park, USA) and stained with 2,3,5-triphenyltetrazolium chloride (TTC; 2%; Sigma-Aldrich, USA) at 37°C and kept in the dark for 30 min, followed by fixation with 10% formalin at room temperature overnight. The infarcted tissue, clearly visible by a lack of TTC staining [14], was outlined on the posterior surface of each slice using an image analyzer (color image scanner, EPSON GT-9000), connected to an image analysis system (AIS software, Imaging research INC, Canada) run on a personal computer, AMD K6-2 3D 400. Infarct volume was obtained as the sum of infarct area per slice multiplied by slice thickness. The infarct volume was expressed as volume (mm^3^) of the whole brain. Both the surgeon and image analyzer operator were blinded to the treatment of each animal.

### Assessment of neurological functions

The modified Bederson score [15] was used 24 h after FC I/R injury to determine global neurological function based on the following scoring system: 0, no deficit; 1, forelimb flexion; 2, decreased resistance to lateral push; 3, unidirectional circling; 4, longitudinal spinning; and 5, no movement.

### Biochemical analysis

Blood was collected from the tail vein for biochemical measurements in experimental rats. Plasma was used for the measurements of total cholesterol and triglyceride (Randox reagent kits, Randox Laboratories LTD. Antrim, United Kingdom). Insulin was measured using a commercial available kit provided by Mercodia (rat insulin ELISA kit, Uppsala, Sweden). Blood glucose levels were determined by the glucose oxidase method (ChemWell ® 2910 Automated EIA and Chemistry Analyzer Spectra, GMI Inc., USA). The NO/ozone chemiluminescence technique (NOA 280; Sievers Instruments; Boulder, CO, USA) was employed for measuring cerebral NO levels. The deproteinized tissue samples were isolated from brain in accordance to previously published procedures with slight modifications [16]. Superoxide production was measured from the cerebrum by lucigenin-enhanced chemiluminescence, using previously described and validated methods [17]. Peroxynitrite production from the cerebrum of different groups of rats was measured by the luminol-enhanced chemiluminescence method as described previously [18].

### Intravital microscopic observation of cremasteric microciculation

The cremaster muscle was prepared for intravital microscopy following methods reported previously [19]. Briefly, animals were anesthetized with pentobarbital (65 mg/kg, i.p.) and surgical exposure of the cremaster muscle. The cremasteric microcirculation was visualized using an intravital microscope (Nikon Measurescope MM-22, Tokyo, Japan). A digital camera (Sony DXC-750 MD, Tokyo, Japan) was adapted between intravital microscope and a videocassette recorder (Sony SVO-9600, Tokyo, Japan) and project the images onto a calibrated monitor (Sony). To minimize variability, four to six venules were selected in each trial and the same section of each venule was observed throughout the experiment. The number of rolling, adherent (attached to the vessel wall for at least 20 s), and transmigrating leukocytes were counted per region off-line during video playback analysis [20]. Both the surgeon and image analyzer operator were blinded to the treatment of each animal.

### Statistics

Data are expressed as mean ± standard error of mean (SEM). Statistical analysis was performed by one-way analysis of variance for combined data and followed by Bonferroni’s tests. P < 0.05 was considered statistically significant.

### Experimental animals

Male Long-Evans rats (age 8 weeks) were purchased from National Lab. Animal Breeding and Research Center (Taipei, Taiwan). The rats were maintained in a climate controlled facility on a 12-h light/12-h dark cycle with free access to water and food. All experimental procedures were conducted in accordance with the *Guide for Care and Use of Laboratory Animals* and approved by the Institutional Animal Care and Use Committee of the Chang Gung University at Taiwan.

## Results

### General characteristics in Non-diabetic control, STZ-diabetic, and insulin-treated diabetic rats

Animals treated with STZ resulted in consistent hyperglycemia and hypoinsulinemia that persisted over the three-week period. At day 21 of the experiment, one week after vehicle or insulin treatment, plasma glucose and insulin levels in diabetic rats were 460 ± 15 mg/dl and 0.56 ± 0.07 μg/l compared with values of 125 ± 5 mg/dl and 2.58 ± 0.36 μg/l in age-matched non-diabetic controls, respectively (*P* < 0.001 and *P* < 0.05; Table 1). As expected, diabetic rats had lower body weights compared with age-matched controls (292 ± 11 vs. 391 ± 7 g, *P* < 0.001; Table 1). By contrast, insulin treatment (4 IU/rat/day) for 1 week significantly attenuated diabetes-induced body weight loss (329 ± 14 vs. 292 ± 11 g, *P* < 0.05), hyperglycemia (153 ± 10 vs. 460 ± 15 mg/dl, *P* < 0.001), and hypoinsulinemia (2.04 ± 0.55 vs. 0.56 ± 0.07 μg/l, *P* < 0.05) (Table 1).

**Table 1:** **Laboratory characteristics in age-matched non-diabetic control (Con), diabetic (DM), and insulin-treated diabetic (DMI) rats before and after FC I/R-injury**

	**Con (n = 10)**	**DM (n = 10)**	**DMI (n = 10)**
Body weight (g)	391 ±7	292 ± 11^*^	329 ± 14^†^
Before FC I/R-injury			
Plasma glucose (mg/dl)	125 ± 5	460± 15^*^	153 ± 10^†^
Plasma insulin (μg/L)	2.58 ± 0.36	0.56 ± 0.07^*^	2.04 ± 0.55^†^
After FC I/R-injury			
Plasma glucose (mg/dl)	194 ± 7	508 ± 43^*^	350 ± 51^†^
Plasma insulin (μg/L)	3.07 ± 0.36	0.61 ± 0.10^*^	1.66 ± 0.41^†^

Data are expressed as mean ± SEM (*P < 0.05 vs. control, ^†^ *P* < 0.05 vs. DM).

### Insulin treatment preserved microvascular functions in STZ-diabetic rats

**Figure 1:**
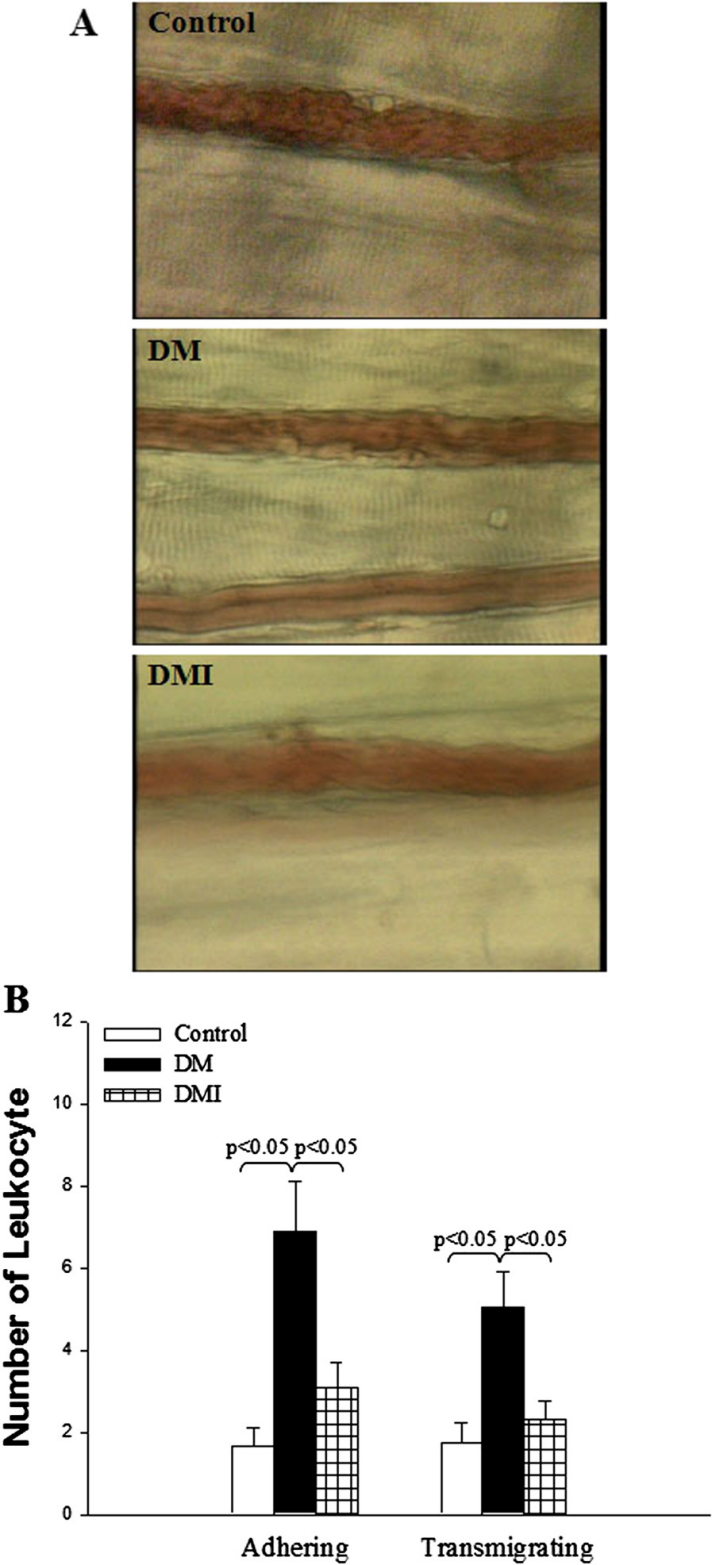
**Diabetes increased leukocyte adherence, and transmigration in the cremasteric postcapillary venules were corrected by insulin treatment.** The numbers of adhering and transmigrating leukocytes per high-power field were counted over a 2-min period. Photographs are shown in **A** and quantified values are shown in **B**. Values are expressed as mean ± SEM (*n* = 15 per each group).

The microvascular functions of cremaster muscles were examined by intravital microscopic observation (Figure 1A). Low rates of leukocyte adherence and transmigrating (1.7 ± 0.44 and 1.7 ± 0.50 numbers/100 μm, respectively) were seen in age-matched non-diabetic controls (Figure 1B). In the STZ-diabetic rats, a large increase of adhering and transmigrating (6.9 ± 1.26 and 5.1 ± 0.85 numbers/100 μm, respectively) leukocytes were observed in the postcapillary venules of the cremaster muscle (Figure 1B). By contrast, DMI had a significantly lower number of adhering (3.1 ± 0.60 numbers/100 μm) and transmigrating (2.3 ± 0.46 numbers/100 μm) leukocytes in the postcapillary venules of the cremaster muscle (Figure 1B).

### Diabetes increased cerebral NO level was corrected by insulin treatment

**Figure 2:**
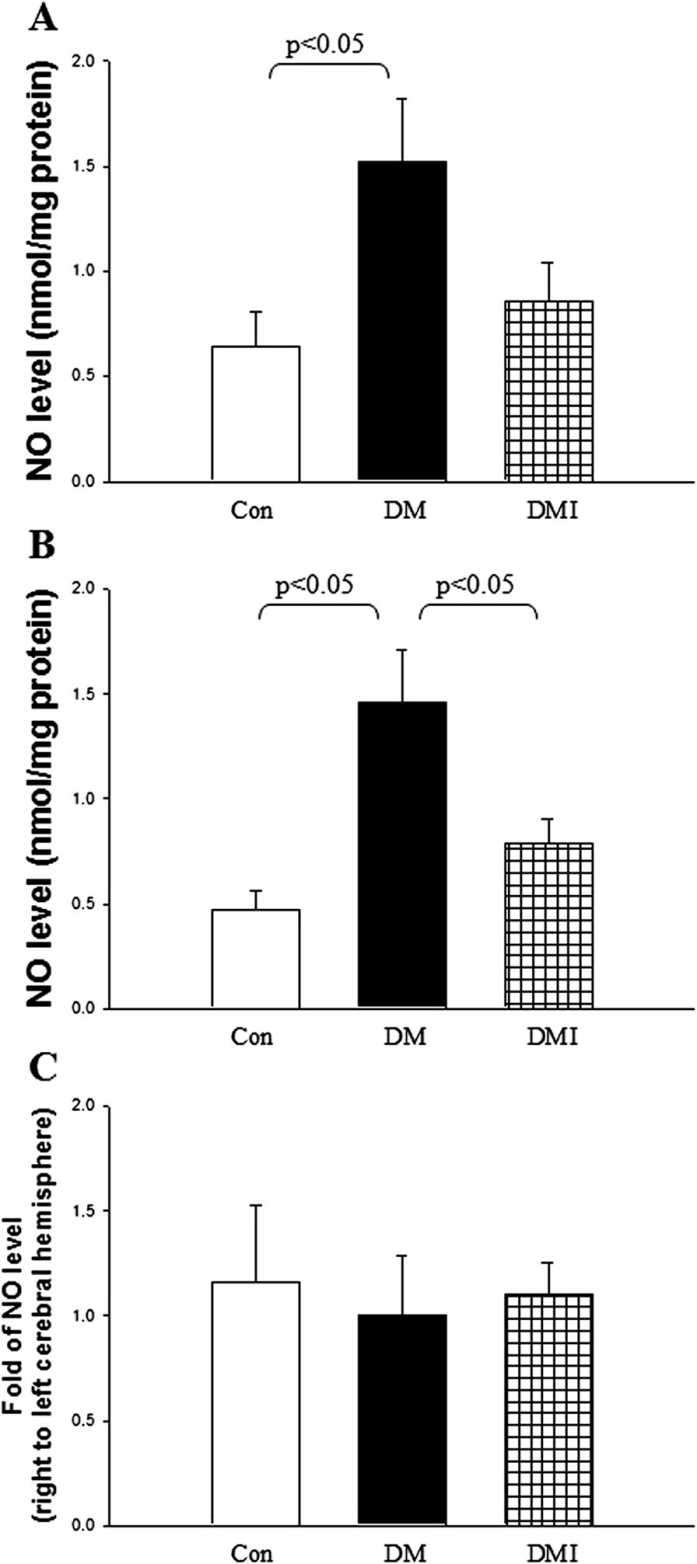
**The cerebral NO levels were compared in control, DM, and DMI rats.** The left **(A)** and right **(B)** cerebral NO levels in rats were evaluated by NO/ozone chemiluminescence technique. **(C)** The ratio of right to left cerebral hemisphere NO level was also evaluated among these groups. Results are expressed as mean ± SEM (*n* = 7 to 8 per each group).

The cerebral NO levels were also examined in control, DM, and DMI rats (Figure 2). Under normoxic conditions, the NO level in the left and right cerebral hemispheres was increased in diabetic rats compared with non-diabetic controls (Figure 2A, B). By contrast, insulin treatment reduced NO levels compared with non-treated diabetic rats (Figure 2A, B). The fold of the NO level in the right to left cerebral hemispheres was no different among the groups (Figure 2C).

### Diabetes aggravated FC I/R-induced brain infarction and neurological deficits were rescued by insulin treatment

**Figure 3:**
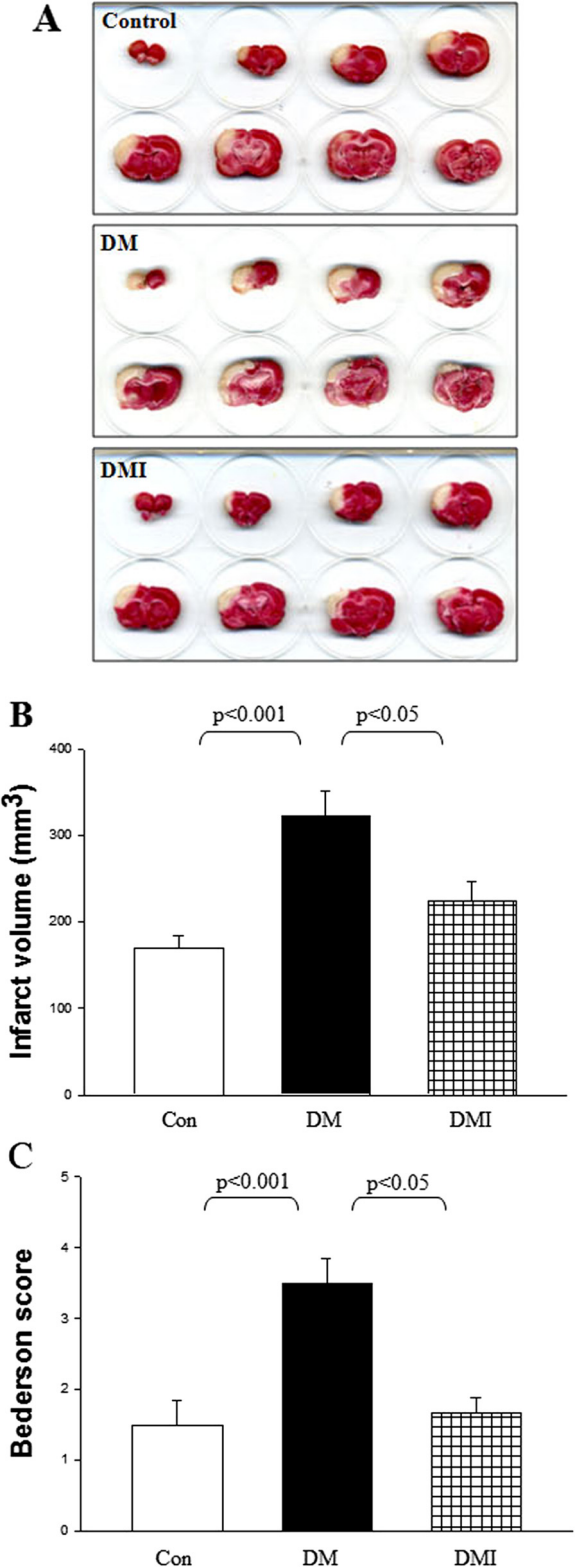
**Diabetes aggravated FC I/R-induced brain infarction and neurological deficits were rescued by insulin treatment.** The diabetic rats were induced by intravenous injection of STZ (65 mg/kg) and then were used 2 weeks later. Insulin (4 IU rat^−1^ day^−1^) was subcutaneously injected in STZ-diabetic rats for 1 week. Infarct volumes were determined after 1-h focal cerebral ischemia followed by 24-h reperfusion. Representative coronal sections **(A)**. Mean ± SEM of quantitative infarct volume **(B)**. The neurological functions were examined by Bederson score **(C)** in age-matched non-diabetic control (*n* = 10), DM (*n* = 10), and insulin-treated diabetic rats (DMI, *n* = 10). Values are expressed as mean ± SEM.

Hyperglycemia and insulin deficiency may have divergent or distinct effects on the progression of ischemic stroke in STZ-diabetic rats. We sought to compare directly the severity of FC I/R-induced infarct volume and neurological deficits (Bederson) in control, diabetic, and insulin-treated diabetic rats. Before FC I/R injury, blood gas parameters (PO_2_, PCO_2_, and pH) had been compared and no statistical difference among the groups (data not shown) was observed. After FC I/R injury, the infarct volume was 170.12 ± 14.42 mm^3^ in the age-matched non-diabetic controls (*n* = 10). STZ-diabetic rats indicated a dramatic enhancement of I/R-induced infarct volume by 1.9-fold compared with non-diabetic controls (*P* < 0.001, *n* = 10). Consistent with its hypoglycemic effect, insulin treatment significantly reduced I/R-induced infarct volume from 321.79 ± 29.41 to 224.91 ± 22.48 mm^3^ (*P* < 0.05, *n* = 10) (Figure 3A, B). For evaluation of neurological functions, Bederson tests were performed to examine the muscle strength and postural reflex, respectively. After ischemic stroke, Bederson scores were 1.5 ± 0.3 in non-diabetic control rats (Figure 3C). The FC I/R-induced muscle strength and postural reflex weakness were further impaired in diabetic rats (3.5 ± 0.3) as compared with non-diabetic controls. By contrast, the diabetic-aggravated neurological deficits were significantly attenuated by insulin treatment (1.7 ± 0.2; Figure 3C).

### General characteristics in Non-diabetic control, STZ-diabetic, and insulin-treated diabetic rats after FC I/R injury

The characteristics of hyperglycemia and hypoinsulinemia did not influence diabetic rats subjected to FC I/R injury. Plasma glucose and insulin levels in diabetic rats were 508 ± 43 mg/dl and 0.61 ± 0.10 μg/l compared with values of 194 ± 7 mg/dl and 3.07 ± 0.36 μg/l in age-matched non-diabetic controls, respectively (*P* < 0.001 and *P* < 0.05; Table 1) 24 hr after cerebral infarction. By contrast, insulin treatment significantly attenuated diabetes-induced hyperglycemia (350 ± 51 vs. 508 ± 43 mg/dl, *P* < 0.05), and hypoinsulinemia (1.66 ± 0.41 vs. 0.61 ± 0.10 μg/l, *P* < 0.01; Table 1). The plasma triglyceride and cholesterol levels were not significantly different among the groups (data not shown).

### Diabetes influenced cerebral NO, superoxide, and peroxinitrite production in FC I/R injury was corrected by insulin treatment

**Figure 4:**
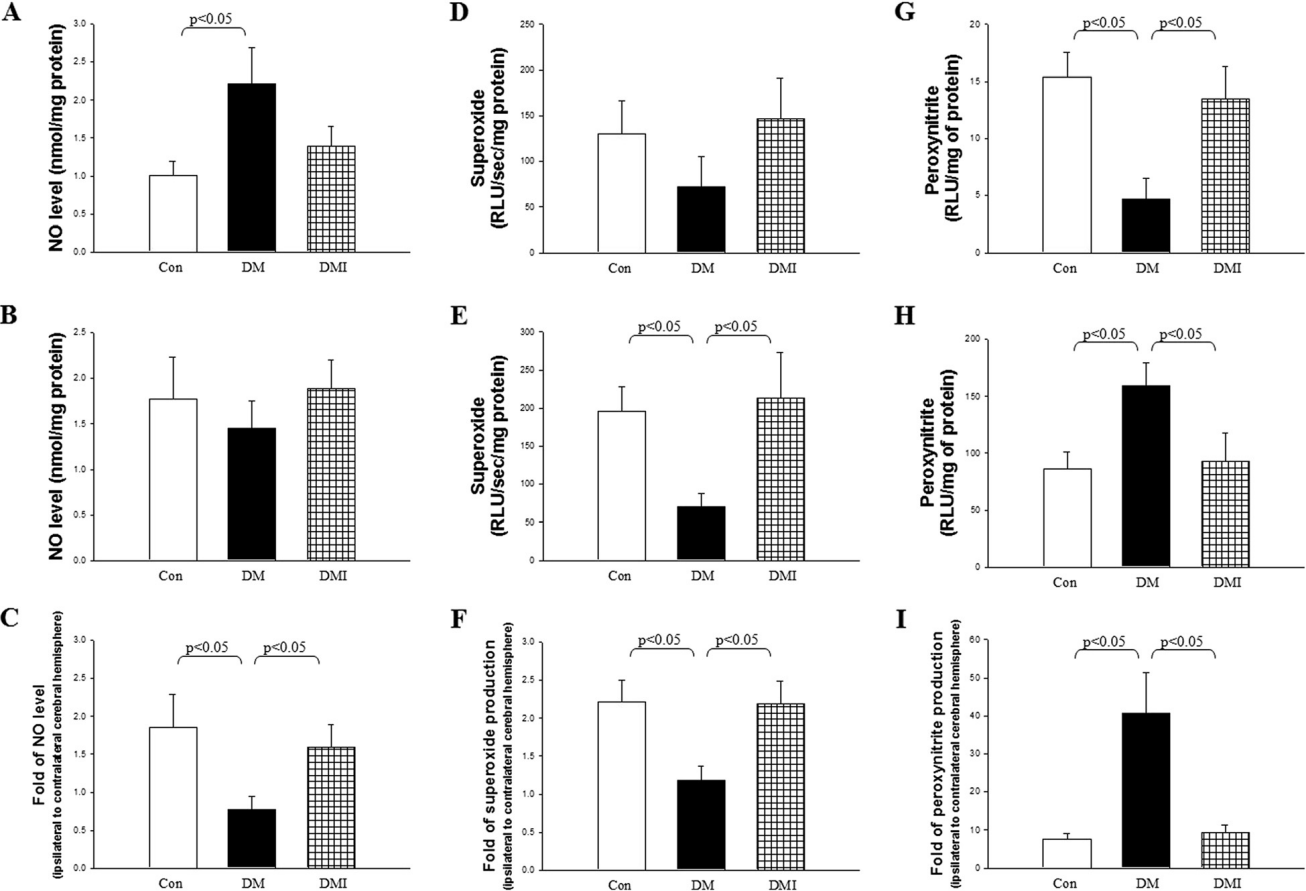
**The cerebral NO, superoxide, and peroxynitrite levels in contralateral and ipsilateral cerebral hemisphere were compared in control, DM, and DMI rats after FC I/R injury.** The contralateral and ipsilateral cerebral hemisphere NO levels **(A and B)**, superoxide anion contents **(D and E)**, and peroxynitrite production **(G and H)** were examined in control, DM, and DMI rats with FC I/R injury. The ratio of ipsilateral to contralateral cerebral NO **(C)**, superoxide **(F)**, and peroxynitrite **(I)** levels was also evaluated among three groups. Results are expressed as mean ± SEM (*n* = 7 to 8 per group).

The cerebral NO, superoxide, and peroxinitrite levels were examined in non-diabetic control, STZ-diabetic, and insulin-treated diabetic rats subjected to FC I/R injury (Figure 4). After FC I/R injury, the variability of NO level in the contralateral cerebral hemisphere of the groups (Figure 4A) was consistent with those rats without FC I/R-injury (Figure 2A, B). However, the variability of NO level was altered in the ipsilateral cerebral hemisphere after FC I/R-injury (Figure 4B) compared with non-FC I/R-injured right cerebral hemisphere (Figure 2B). The fold of ipsilateral to contralateral cerebral hemisphere NO level was significantly reduced in FC I/R-injured diabetic rats compared with non-diabetic controls (Figure 4C). By contrast, insulin treatment significantly increased the fold of ipsilateral to contralateral cerebral hemisphere NO level (Figure 4C).

Among these three groups, we observed no significant difference in the contralateral cerebral hemisphere superoxide levels after FC I/R injury (Figure 4D). However, superoxide level in the ipsilateral cerebral hemisphere was significantly decreased in diabetic rats compared with age-matched controls after FC I/R injury. Insulin treatment significantly reversed diabetes-induced cerebral superoxide level loss (Figure 4E). The fold of ipsilateral to contralateral cerebral hemisphere superoxide level was significantly reduced in FC I/R-injured diabetic rats compared with non-diabetic controls (Figure 4F). The diabetes-induced superoxide level decrease was significantly reversed by insulin treatment (Figure 4F).

After FC I/R injury, in the contralateral cerebral hemisphere, diabetes significantly reduced peroxynitrite levels compared with non-diabetic controls, whereas treatment with insulin showed a significantly higher peroxynitrite levels compared with the non-treated diabetic group (Figure 4G). By contrast, peroxynitrite level in the ipsilateral cerebral hemisphere was significantly increased in diabetic rats compared with age-matched controls after FC I/R injury. Insulin treatment significantly reversed diabetes-induced cerebral peroxynitrite production (Figure 4H). Interestingly, the peroxynitrite level in ipsilateral cerebral hemisphere was 40-fold to contralateral cerebral hemisphere in STZ-diabetic rats. After FC I/R injury, diabetic rats showed a dramatic enhancement of peroxynitrite production compared with non-diabetic controls, and the diabetes-induced peroxynitrite level increase was significantly attenuated by insulin treatment (Figure 4I).

## Discussion and conclusions

The primary findings of the present study are as follows: (1) Hyperglycemia exacerbated microvascular functions and increased cerebral NO production. (2) Hyperglycemia exacerbated FC I/R-induced infarct volume and neurological dysfunction. (3) Parallel with its hypoglycemic effects, insulin also improved microvascular functions and attenuated FC I/R injury in STZ diabetes. (4) Diabetes increased NO reaction with superoxide to form peroxinitrite in the cerebrum after FC I/R injury, and this effect was reversed by insulin treatment.

The present study shows that diabetes increased leukocyte adherence and transmigration in the postcapillary venules of rat cremaster muscle. The cremaster muscle is a powerful model for direct evaluation of microvascular function in vivo. However, the influence of diabetes on leukocyte adherence and transmigration in the postcapillary venules of rat cremaster muscle have yielded conflicting results. Bonnardel-Phu and Vicaut reported that no difference was found in adherent leukocytes or in the leukocyte rolling flux between diabetic and normoglycemic rats [21]. In contrast, Pettersson et al., reported that sustained hyperglycemia results in increased levels of adherent and emigrated leukocytes in mouse models of type 1 and type 2 diabetes [22]. In this study, our results show the impairment of microvascular functions in STZ diabetes. However, the administration of insulin greatly lowered the plasma glucose level, restored the microvascular dysfunction, and rescued FC I/R injury in STZ-diabetic rats.

Oxidative stress, an imbalance between free radical generation and elimination or detoxification, is a principal mediator of cerebral injury during ischemia and reperfusion. In addition, oxidative stress is known to be involved in the development and progression of diabetes and diabetic complications [23]. The unifying pathophysiological mechanism that underlies ischemic stroke in diabetes could be explained by increased production of reactive oxygen species (ROS) and nitrogen species (RNS) [1]. However, studies of diabetic NO levels have yielded conflicting results [5-8]. In this study, we showed that diabetes increased NO production which was reversed by insulin treatment under normoxic conditions. A dominant paradigm to explain the increase of NO in diabetes is that a T lymphocyte-dependent autoimmune process activates macrophages or β-cells [24] result from the decreased production of TGF-β1 and increased expression of iNOS pathway by macrophage. In contrast, insulin administration to STZ-induced diabetic rodents decreases macrophage NO production by down-regulation of the iNOS pathway [25]. Evidence suggests that there is an increase in the activity of inducible nitric oxide (iNOS) in diabetes. The increased NO production in diabetic brain with subsequent development of local oxidative stress is supposed to be the possible pathophysiological mechanism to impair endothelium-dependent relaxation of intracranial blood vessels during STZ-induced diabetes [26]. The FC I/R injury also increased NO production by 3.7-fold in non-diabetic control rats. Under normoxic condition, the fold of right to left cerebral hemisphere NO level was not significantly different among the groups. By contrast, the fold of ipsilateral to contralateral cerebral hemisphere NO levels was significantly reduced in FC I/R-injured diabetic rats and treatment with insulin rescued the above phenomenon. These results indicate that both diabetes and FC I/R injury increased cerebral NO production, however, diabetes-aggravated FC I/R injuries may be related, with loss of efficacy of the stimulated NO produced. These effects were reversed by insulin treatment.

Furthermore, we found that superoxide level in the ipsilateral cerebral hemisphere and in the fold of ipsilateral to contralateral cerebral hemisphere superoxide levels were significantly decreased in FC I/R-injured diabetic rats compared with non-diabetic controls. The diabetes-induced superoxide level decrease was significantly reversed by insulin treatment. These results indicate that diabetic rats reduced cerebral superoxide levels after FC I/R injury. By contrast, insulin treatment significantly reversed the decrease of superoxide levels.

All ROS and RNS were previously considered toxic agents capable of damaging biomolecules. However, physiological free radicals superoxide and nitric oxide are recently known to be relatively harmless species but are able to mediate or initiate many enzyme- and gene-dependent reactions in both physiological and pathophysiological processes [27]. NO may react with superoxide to form reactive peroxynitrite (ONOO–). Peroxynitrite is a potent oxidizing agent that can cause DNA fragmentation and lipid peroxidation [28]. Multiple lines of evidence demonstrating the formation of peroxynitrite in diabetic vasculature exist, both in experimental animals and in humans. One of the important pathways of peroxynitrite-mediated vascular dysfunction in diabetes involves activation of the nuclear enzyme poly(ADP-ribose) polymerases (PARP). Peroxynitrite-induced overactivation of PARP consumes NAD + and consequently ATP culminating in cell dysfunction, apoptosis, or necrosis [29].

Our results show that diabetes slight decreased superoxide anion production but significantly decreased peroxynitrite contents in the contralateral brain region. Many studies demonstrated that endothelium-derived NO was greatly reduced under diabetic state [30,31]. Our previous study also shows that phosphorylated eNOS protein level was dramatic decreased in the brain of FC I/R diabetic rats [32]. Since peroxynitrite is formed by the NO-superoxide reaction, reduction of superoxide anion and endothelium-derived NO might be a possible reason for lower peroxynitrite production in the contralateral brain region of FC I/R diabetic rats.

In addition, we also shows that diabetes slight decreased NO level but significantly increased peroxynitrite production in the ipsilateral brain region of FC I/R diabetic rats. Our previous study also shows that phosphorylated eNOS protein level was dramatic decreased in brain of FC I/R diabetic rats [32]. This may cause eNOS uncoupling and consequently increase free radical production and decrease endothelium-derived NO. Under this condition, most NO can react with superoxide to form peroxynitrite [33].

In this study, interestingly, although the peroxinitrite level in the contralateral cerebral hemisphere was decreased in diabetic rats compared with that in non-diabetic controls after FC I/R injury and insulin significantly rescued the phenomenon, however, the level of peroxynitrite in the contralateral cerebral hemisphere was very few compared with the amount in the ipsilateral cerebral hemisphere. By contrast, peroxynitrite level in the ipsilateral cerebral hemisphere and in the fold of ipsilateral to contralateral cerebral hemisphere peroxynitrite levels were significantly increased in diabetic rats compared with age-matched controls after FC I/R injury. Insulin treatment significantly reversed the diabetes-induced cerebral peroxynitrite production. Interestingly, the peroxynitrite level in ipsilateral cerebral hemisphere was 40-fold to contralateral cerebral hemisphere in STZ-diabetic rats. After FC I/R injury, diabetic rats showed a dramatic enhancement of peroxynitrite production compared with non-diabetic controls, and the diabetes-induced peroxynitrite level increase was significantly attenuated by insulin treatment.

These results indicate that diabetes stimulated NO production and descreased superoxide production and peroxynitrite formation in normoxic brain region (contralateral brain area). However, the formation of peroxynitrite in diabetic rats increased significantly in I/R brain region (ipsilateral brain area) which may contribute to the prominent increase in brain injury. The increase in peroxynitrite formation and brain injury can be alleviated by insulin treatment.

In conclusion, glycemic control with insulin treatment may have clinical benefits for stroke patients with diabetes, especially in those with insulinopenic diabetes. Our results show that NO/superoxide/peroxynitrite formation and its normalization contribute to detrimental and protective effects of diabetes and insulin, respectively, in the context of ischemic stroke. Finally, our findings indicate that insulin renders diabetic rats resistant to acute ischemic stroke by arresting NO reaction with superoxide to form peroxynitrite.
